# Dietary Fermented Blueberry Pomace Supplementation Improves Small Intestinal Barrier Function and Modulates Cecal Microbiota in Aged Laying Hens

**DOI:** 10.3390/ani14192786

**Published:** 2024-09-26

**Authors:** Binghua Qin, Zhihua Li, Qian Zhu, Ting Chen, Wei Lan, Yadong Cui, Md. Abul Kalam Azad, Xiangfeng Kong

**Affiliations:** 1 Key Laboratory of Agro-Ecological Processes in Subtropical Region, Hunan Provincial Key Laboratory of Animal Nutrition Physiology and Metabolic Processes, Institute of Subtropical Agriculture, Chinese Academy of Sciences, Changsha 410125, China; qinbinghua23@mails.ucas.ac.cn (B.Q.); zhli92@126.com (Z.L.); zhuqian@isa.ac.cn (Q.Z.); chenting23@mails.ucas.ac.cn (T.C.); 2 School of Biology and Food Engineering, Fuyang Normal University, Fuyang 236037, China; lanwei@fynu.edu.cn (W.L.); fysyswx@163.com (Y.C.); 3 College of Advanced Agricultural Sciences, University of Chinese Academy of Sciences, Beijing 100049, China

**Keywords:** aged hens, feed additive, fruit pomace, late laying period, microbiota, small intestine

## Abstract

**Simple Summary:**

The use of fermented agricultural by-products as novel feed additives to improve animal health and livestock production has attracted attention due to their enhanced nutritional values after fermentation. Blueberry pomace, rich in bioactive substances, has health benefits. This study investigated the effects of fermented blueberry pomace on intestinal health in late-phase laying hens. The findings indicated that dietary fermented blueberry pomace supplementation could improve small intestinal barrier function and modulate cecal microbiota in laying hens during the late laying period and thus could be a potential feed additive in poultry production.

**Abstract:**

This study aimed to investigate the effects of fermented blueberry pomace (FBP) on the intestinal barrier function and cecal microbiome of aged laying hens. A total of 320 Yukou Jingfen No. 8 laying hens (345-day-old) were randomly divided into a control group, 0.25% FBP group, 0.5% FBP group, or 1.0% FBP group. The results showed that the villus height (VH) in the jejunum of the 0.25–0.5% FBP groups and villus surface area in the jejunum of the 0.25% FBP group were higher (*p* < 0.05), while 0.25% FBP supplementation displayed a higher (*p* = 0.070) VH in the ileum compared to the control group. *Mucin-2* expression was upregulated (*p* < 0.05) in the jejunum of the 0.5% FBP group and the ileum of the 0.25–0.5% FBP groups. Compared to the control group, interleukin (*IL*)-4 and *IL-13* expressions were upregulated (*p* < 0.05) in the 1.0% FBP group. Microbiota analysis revealed that *Prevotella* abundance in the cecum of the 0.5–1.0% FBP groups was higher (*p* < 0.05) than in the 0.25% FBP group. In addition, microbial function prediction analysis showed that cecal microbiota in the 0.25% FBP group were mainly enriched by alanine/aspartate/glutamate metabolism and methane metabolism. Moreover, Spearman’s correlation analysis revealed the potential correlations between the abundance of the cecal microbiota and intestinal-barrier-function-related gene expressions, as well as the short-chain fatty acid content, of laying hens. In summary, dietary FBP supplementation enhanced intestinal barrier function by improving intestinal morphology, upregulating gene expressions related to barrier function, and altering the cecal microbiota of aged laying hens.

## 1. Introduction

The intestine is not only an important site for the digestion and absorption of nutrients but also plays an important role in the immune system in poultry [1]. The intestine is home to a complex community of microbes, which play an important role in regulating host health [2]. The gut microbiota plays a crucial role in the absorption and utilization of nutrients, synthesis of vitamins, and regulation of gut structure and microbial composition [3]. Dysbiosis of the gut microbiota composition of aged laying hens leads to lower production performance and diseases such as chronic inflammation [4]. Therefore, maintaining intestinal health is of great significance for maintaining laying hens’ health and ensuring production efficiency.

The misuse and overuse of antibiotic growth promoters in animal production have contributed to the rise and spread of multidrug-resistant bacteria, which could transfer from animals to humans and have severe public health implications [5]. Therefore, scholars are searching for greener and safer novel feed additives as antibiotic alternatives. Recently, fruit pomace has gained more interest as a novel feed additive. Additionally, studies have focused on the effects of fruit pomace on the production performance, egg quality, and intestinal health of laying hens because pomace contains abundant bioactive components. Selim et al. [6] showed that supplementing 90 g/kg grape pomace to a laying hen’s diet during the peak laying period improved production performance by increasing the laying rate and weight and improved egg quality by enriching the yolk with beneficial fatty acids and enhancing the antioxidant capacity of yolk lipids. Moreover, Jackson et al. [7] found that 6% apple pomace supplementation enhanced the intestinal health of laying hens by increasing the villus area and Paneth cell number and improving the microbial community. The blueberry, known as the king of berries, is a deciduous shrub of the genus *Vaccinium* (family Ericaceae) with high nutritional value and health benefits. Blueberry pomace has attracted wide attention due to its rich bioactive substances, including anthocyanins, polyphenols, and flavonoids [8]. A previous study found that 4% whole blueberry powder enhanced intestinal immune function by downregulating inflammatory gene expressions in high-fat-diet-induced mice [9]. However, the high moisture content and antinutritional factors present in fruit pomace prevent its effective application as a poultry feed additive.

Microbial fermentation not only reduces moisture by adding excipients, ultimately solving the storage and transportation problems, but also improves the nutritional structure of fruit pomace by improving probiotic activity, transforming plant-based proteins, and degrading antinutritional factors. Recent studies have reported that fermented blueberry pomace (FBP) could reduce the obesity and inflammation of high-fat mice and provide potential value for human health [10]. However, little information exists on the effects of dietary FBP supplementation in laying hens. Our previous study showed that dietary FBP supplementation could improve the egg quality and nutritional value of laying hens during the late laying period [11]. Considering the potential effects of FBP, we hypothesized that supplementing FBP to laying hens’ diet would effectively enhance intestinal health by improving intestinal morphology, enhancing intestinal barrier function, and altering the microbiota community. Therefore, the present study aimed to investigate the effects of FBP on the intestinal barrier function and cecal microbiota composition of aged laying hens.

## 2. Materials and Methods

### 2.1. FBP Preparation

Dried blueberry pomace was provided by Anhui Xiuqin Agricultural Technology Co. Ltd. (Fuyang, China). The fermentation processes and conditions were consistent with our previous study [11]. In addition, the composition of the routine nutrients, amino acids, and fatty acid contents in FBP was determined according to the methods described in our previous study [11].

### 2.2. Birds and Feeding Management

A total of 320 Yukou Jingfen No. 8 laying hens (345-day-old) with similar body weights were selected and randomly assigned into four treatments. Each treatment contained eight replicates with ten hens per replicate. After seven days of the adaptation period, the birds in the control group were fed a basal diet without FBP, while the birds in the treatment groups were fed a basal diet supplemented with 0.25% FBP, 0.5% FBP, and 1.0% FBP on top of the diet, respectively. The formal experiment lasted 56 days. Five birds were housed in one cage and had free access to feed and water. The cages were located in a well-ventilated room, maintaining a temperature of 18–23 °C, humidity of 55–65%, and light/dark cycles of 16/8 h. The composition and nutrient levels of the basal diet are presented in Table 1.

### 2.3. Sample Collection

On day 56 of the trial, one hen from each replicate (eight hens per group), close to the average body weight per replicate, was randomly selected for sampling. After 12 h of fasting, the hens were euthanized by bleeding the neck. Samples of two parts of the jejunum and ileum (middle position) were collected; one part was fixed in 4% paraformaldehyde for morphology analysis, and the other part was frozen in liquid nitrogen and stored at –80 °C for gene expression analysis. The cecal contents (2 cm in length, middle position) were collected, immediately frozen in liquid nitrogen, and stored at –80 °C for microbiota composition and metabolite analyses.

### 2.4. Intestinal Morphology Analysis

Jejunal and ileal samples fixed in 4% paraformaldehyde were dehydrated, cleared in xylene, waxed, and embedded in paraffin. The intestinal samples were then cut into 5 µm thick slices, stained with hematoxylin and eosin (H&E), dyed, and sealed. Villus height (VH), villus width (VW), and crypt depth (CD) of the jejunum and ileum were measured using an optical microscope (Nikon ECLIPSE 80i, Tokyo, Japan). The VH to CD ratio (VCR) and villus surface area (VSA) were calculated using the following equation [12]:

(1) $$VCR=\frac{VH}{CD}$$

(2) $$VSA=\pi \times \frac{VW}{2}\times \sqrt{\left[{\left(\frac{VW}{2}\right)}^{2}+{VH}^{2}\right]}$$

### 2.5. Intestinal Barrier Function Gene Expression

The total RNA of the jejunum and ileum was extracted with the AG RNAex Pro reagent (Accurate Biology, Changsha, China) following the manufacturer’s instructions. The purity and concentration of the extracted RNA were validated using a NanoDrop ND-2000 spectrophotometer (Thermo Fisher Scientific, Waltham, MA, USA). Then, the extracted total RNA was reverse-transcribed into cDNA using an Evo M-MLV RT kit (Accurate Biology). The real-time quantitative PCR (RT-qPCR) analysis was performed on a LightCycler^®^ 480II Real-Time PCR System (Roche, Basel, Switzerland) with SYBR^®^ Green Premix Pro Taq HS qPCR Kit (Accurate Biology). The RT-qPCR was performed in a total reaction volume of 10 µL, including 1 µL cDNA, 0.4 µL each for forward and reverse primers, 5 µL SYBR^®^ Green Premix (Accurate Biology), and 3.2 µL ddH_2_O. The RT-qPCR reaction conditions were as follows: an initial denaturation at 95 °C for 30 s, followed by 45 cycles of denaturation at 95 °C for 10 s and annealing at 53 °C for 10 s, and a final extension at 72 °C for 20 s. The primer sequences are presented in Table 2. The relative mRNA expression for each gene was calculated using the 2^–ΔΔCt^ method [13].

### 2.6. Cecal Microbiota Analysis

Microbial DNA from the individual cecal contents was extracted using a Fast DNA SPIN extraction kit (MP Biomedicals, Santa Ana, CA, USA) according to the manufacturer’s instructions. The V3–V4 hypervariable regions of the microbial 16S rRNA gene were amplified using PCR with the forward primer F (5′-ACTCCTACGGGAGGCAGCA-3′) and the reverse primer R (5′-GGACTACHVGGGTWTCTAAT-3′). Illumina MiSeq sequencing and bioinformatics analysis were performed by the Personal Biotechnology Co., Ltd. (Shanghai, China) on an Illumina NovaSeqPE 250 platform (Illumina, San Diego, CA, USA).

Quantitative Insights into Microbial Ecology 2 (QIIME2) was used to process the sequences, and DADA2 was used to determine amplicon sequence variants (ASVs) through denoising. The taxonomy and annotation of ASVs were based on the Greengenes database. The diversity plugin was used to determine the alpha-diversity indices, including Chao1, Observed_species, Shannon index, Simpson index, Pielou-evenness, Faith’s Phylogenetic Diversity, and Goods coverage. A non-metric multidimensional scaling (NMDS) map based on the Bray–Curtis distance was used to determine beta-diversity. Taxonomic distributions at the phylum and genus levels were determined using a Kruskal–Wallis test. A linear discriminant analysis (LDA) effect size (LEfSe) analysis was performed to reveal the biomarkers among the different groups. Phylogenetic Investigation of Communities by Reconstruction of Unobserved States (PICRUSt2) software (https://github.com/picrust/picrust2, accessed on 5 July 2024) was used to predict functional gene composition. The Kyoto Encyclopedia of Genes and Genomes (KEGG) pathway and function abundances were calculated according to the KEGG database.

### 2.7. Short-Chain Fatty Acid (SCFA) Analysis

The SCFA contents were determined based on our previously described method [14]. Briefly, cecal contents (0.5 g) were weighed and suspended in 2.5 mL of ddH_2_O overnight at 4 °C. After centrifuging at 10,000× *g* for 10 min, the supernatants were collected and thoroughly mixed with 2 mL ddH_2_O for 30 min. Then, the mixtures were centrifuged, and the supernatants were transferred to 5 mL colorimetric tubes. After centrifuging at 12,000× *g* and 4 °C for 15 min, 25% of metaphosphoric acid was added to the supernatants. Finally, the supernatants were filtered through a 0.45 µm filter membrane and analyzed using gas chromatography (7890A, Agilent, Santa Clara, CA, USA).

### 2.8. Statistical Analysis

All data were analyzed using SPSS 26.0 software (IBM Inc., Chicago, IL, USA). The means and comparisons among different treatment groups were examined by a one-way ANOVA and Tukey’s *post-hoc* test. The correlation between intestinal-barrier-function-related gene expressions, as well as SCFA concentrations and cecal microbiota abundances at the phylum and genus levels, was determined using Spearman’s correlation by the genescloud tool, a free online platform for data analysis (https://www.genescloud.cn, accessed on 5 July 2024). All data are presented as the mean and standard error of the mean (SEM). Data were considered statistically significant if *p* < 0.05, and trends if 0.05 ≤ *p* < 0.10.

## 3. Results

### 3.1. Effects of FBP on the Morphology of the Jejunum and Ileum in Laying Hens

**Figure 1:**
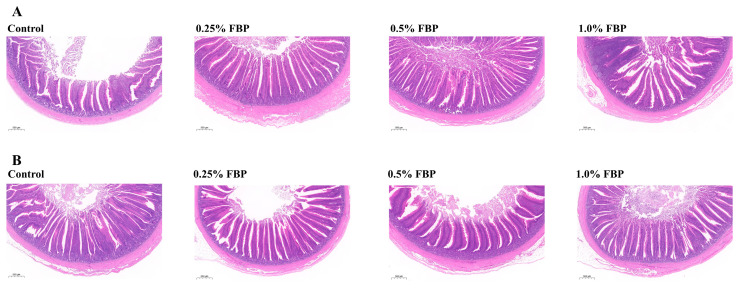
Effects of fermented blueberry pomace (FBP) on the jejunal (**A**) and ileal (**B**) morphology of laying hens during the late laying period. Scale bars, 500 μm (magnification 20×).

The effects of FBP on the intestinal morphology of laying hens during the late laying period are shown in Table 3 and Figure 1. Dietary 0.25% and 0.5% FBP supplementation increased (*p* < 0.05) the VH in the jejunum, whereas 0.25% FBP increased (*p* < 0.05) the VSA in the jejunum of laying hens compared to the control group. Dietary 0.25% FBP supplementation displayed an increasing trend (*p* = 0.070) in the ileal VH (Table 3). According to the results of H&E, the villi structure in the jejunum and ileum of laying hens was uniform and regularly aligned in the FBP groups compared to the control group (Figure 1). No significant differences (*p* > 0.05) in the CD, VCR, and VW in the jejunum and ileum and VSA in the ileum were observed among different groups (Table 3).

### 3.2. Effects of FBP on Barrier-Function-Related Gene Expressions in the Jejunum and Ileum of Laying Hens

**Figure 2:**
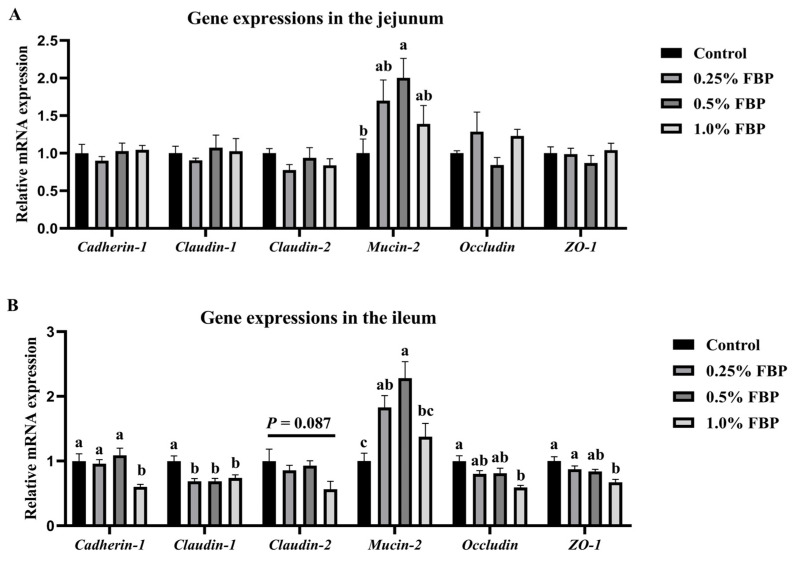
Effects of fermented blueberry pomace (FBP) on the intestinal-barrier-function-related gene expressions in the jejunum (**A**) and ileum (**B**) of laying hens. Data are expressed as the mean and SEM (*n* = 8). Different lowercase letters denote a significant difference (*p* < 0.05). *ZO-1*, zonula occludens-1.

Gene expressions related to the intestinal barrier function of laying hens during the late laying period are shown in Figure 2. *Mucin-2* expression was upregulated (*p* < 0.05) in the jejunum of the 0.5% FBP group, as well as in the ileum of the 0.25–0.5% FBP groups, when compared to the control group. *Cadherin-1*, *occludin*, and *zonula occludens-1* (*ZO-1*) expressions in the ileum of the 1.0% FBP group and *claudin-1* expression in the ileum of the 0.25–1.0% FBP groups were downregulated (*p* < 0.05), while the dietary 1.0% FBP group displayed a downregulating trend in the *claudin-2* expression (*p* = 0.087) in the ileum compared to the control group. No significant differences in the expressions of mechanical-barrier-function-related genes in the jejunum, including *cadherin-1*, *claudin-1*, *claudin-2*, *occludin*, and *ZO-1*, were observed between the control and FBP groups (*p* > 0.05).

**Figure 3:**
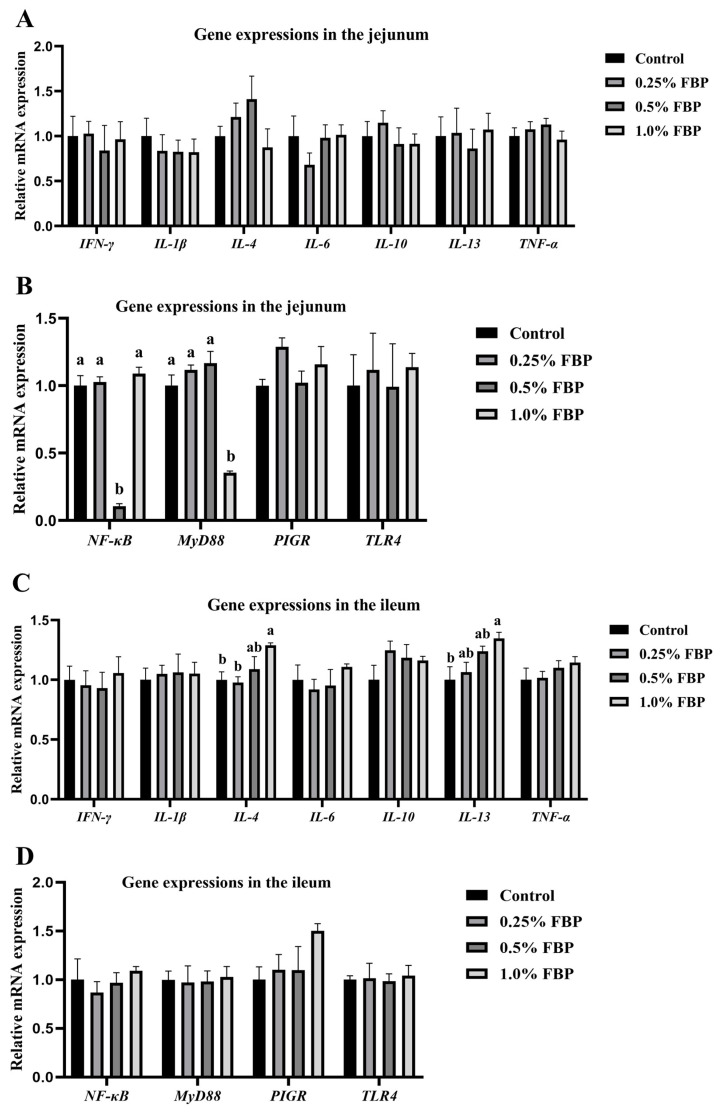
Effects of fermented blueberry pomace (FBP) on the intestinal-barrier-function-related gene expressions in the jejunum (**A**,**B**) and ileum (**C**,**D**) of laying hens during the late laying period. Data are expressed at the mean and SEM (*n* = 8). Different lowercase letters denote a significant difference (*p* < 0.05). *IFN-γ*, interferon gamma; *IL-1β*, interleukin 1 beta; *IL-4*, interleukin 4; *IL-6*, interleukin 6; *IL-10*, interleukin 10; *IL-13*, interleukin 13; *MyD88*, myeloid differentiation factor 88; *NF-κB*, nuclear factor kappa-B; *PIGR*, polymeric immunoglobulin receptor; *TLR4*, toll-like receptor 4; *TNF-α*, tumor necrosis factor alpha.

The effects of FBP on the intestinal-barrier-function-related gene expressions in the jejunum and ileum of laying hens during the late laying period are shown in Figure 3. No significant differences (*p* > 0.05) were observed in the expressions of intestinal-barrier-function-related genes in the jejunum and ileum, including polymeric immunoglobulin receptor (*PIGR*), tumor necrosis factor-alpha (*TNF-α*), interferon-gamma (*IFN-γ*), interleukin (*IL*)*-1β*, *IL-6*, *IL-10*, and toll-like receptor 4 (*TLR4*), between the control and FBP groups (Figure 3A–D). The jejunal nuclear factor kappa-B (*NF-κB*) expression of the 0.5% FBP group and myeloid differentiation factor 88 (*MyD88*) expression of the 1.0% FBP group were downregulated (*p* < 0.05) compared to the control group (Figure 3B). Dietary 1.0% FBP supplementation upregulated (*p* < 0.05) the *IL-4* and *IL-13* expressions in the ileum of laying hens compared to the control group (Figure 3C).

### 3.3. Effects of FBP on SCFAs in the Cecum of Laying Hens

The effects of FBP on SCFA concentrations in the cecum of laying hens are shown in Table 4. No significant differences were observed in the concentrations of SCFAs, including acetic acid, butyric acid, isobutyric acid, isovaleric acid, propionic acid, and valeric acid, between the control and FBP groups (*p* > 0.05).

### 3.4. Effects of FBP on the Cecal Microbiota Profile and Function Prediction of Laying Hens

**Figure 4:**
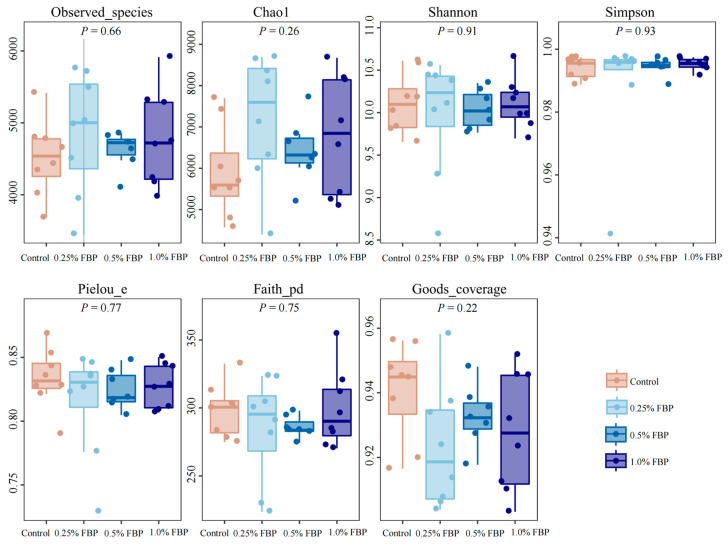
Effects of fermented blueberry pomace (FBP) on the alpha-diversity indices of the cecal microbiota of laying hens during the late laying period (*n* = 8).

**Figure 5:**
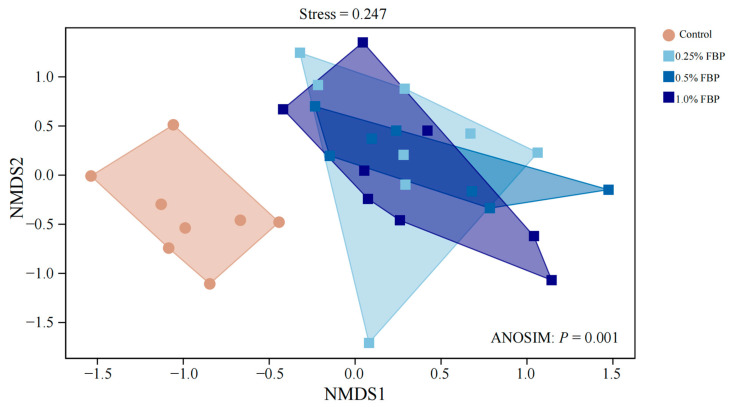
Effects of fermented blueberry pomace (FBP) on the beta-diversity indices of the cecal microbiota of laying hens during the late laying period (*n* = 8).

There were no significant differences (*p* > 0.05) in the Observed-species, Chao1, Shannon, Simpson, Pielou’s, Faith-pd, and Goods coverage indices between the control group and FBP groups (Figure 4). However, principal coordinate analysis (PCoA, Figure 5) showed a distinct separation of the cecal microbial communities between the control and FBP groups.

**Figure 6:**
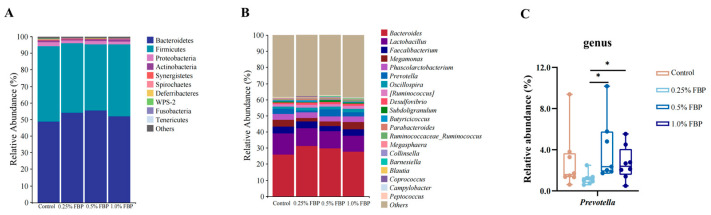
Effects of fermented blueberry pomace (FBP) on microbial community composition in the cecum of laying hens during the late laying period (*n* = 8): the relative abundance of cecal microbiota at the phylum (**A**) and genus (**B**) levels and taxonomic differences among different groups at the genus level (**C**). * *p* < 0.05.

As shown in Figure 6, at the phylum level, *Bacteroidetes* and *Firmicutes* were the most dominant phyla, followed by *Proteobacteria* and *Actinobacteria*, while the abundances of other bacterial phyla were relatively lower (Figure 6A). At the genus level, *Bacteriodies* and *Lactobacillus* were the most dominant genera, followed by *Faecalibacterium*, *Megamonas*, *Phascolarctobacterium*, *Prevotella*, and *Oscillospira* (Figure 6B). A Kruskal–Wallis test was used to further identify the differences in cecal microbial communities at the genus level. As shown in Figure 6C, the relative abundance of *Prevotella* was higher (*p* < 0.05) in the 0.5 and 1.0% FBP groups compared to the 0.25% FBP group.

**Figure 7:**
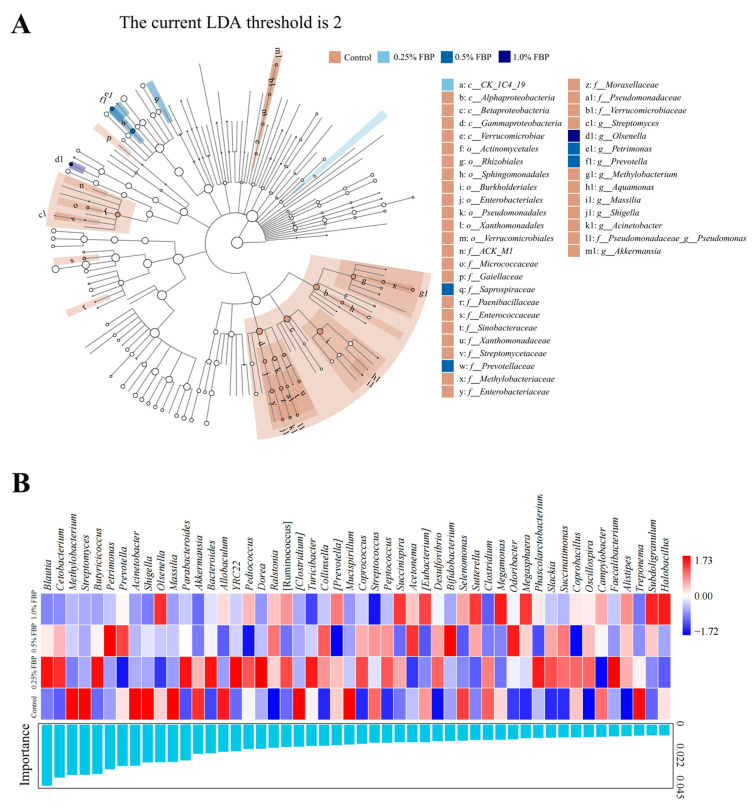
Effects of fermented blueberry pomace (FBP) on the differential bacterial taxa in the cecum of laying hens during the late laying period (*n* = 8): (**A**) taxonomic cladogram and (**B**) histogram of the distribution of LDA values for different species identified by the LEfSe algorithm.

To further identify the differences in cecal microbiota communities among the different groups, the LEfSe analysis (LDA threshold ≥ 2) was performed at the genus level. Eight bacterial biomarkers, including *Methylobacterium*, *Aquamonas*, *Massilia*, *Shigella*, *Acinetobacter*, *Pseudomonas*, *Akkermansia*, and *Streptomyces*, were enriched in the control group. Additionally, *Petrimonas* and *Prevotella* were enriched in the 0.5% FBP group, while *Olsenella* was enriched in the 1.0% FBP group (Figure 7A). As shown in Figure 7B, the top five biomarkers causing differences between the control and FBP groups were *Blautia*, *Cetobacterium*, *Methylobacterium*, *Streptomyces*, and *Butyricicoccus*.

**Figure 8:**
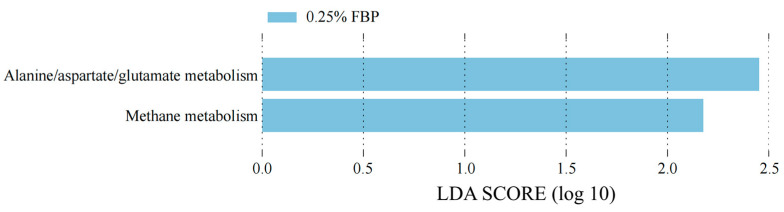
Differences in metabolic functions based on PICRUSt2 analysis of the cecal microbiota of laying hens during the late laying period (*n* = 8).

The KEGG pathways at level 3 predicted by PICRUSt2 were used to understand the microbial metabolic function in the cecum of laying hens during the late laying period (Figure 8). Dietary 0.25% FBP supplementation enriched (*p* < 0.05) the relative abundances of microbial genes involved in alanine/aspartate/glutamate metabolism and methane metabolism.

### 3.5. Correlation between the Gene Expressions, SCFAs, and Cecal Microbiota of Laying Hens

**Figure 9:**
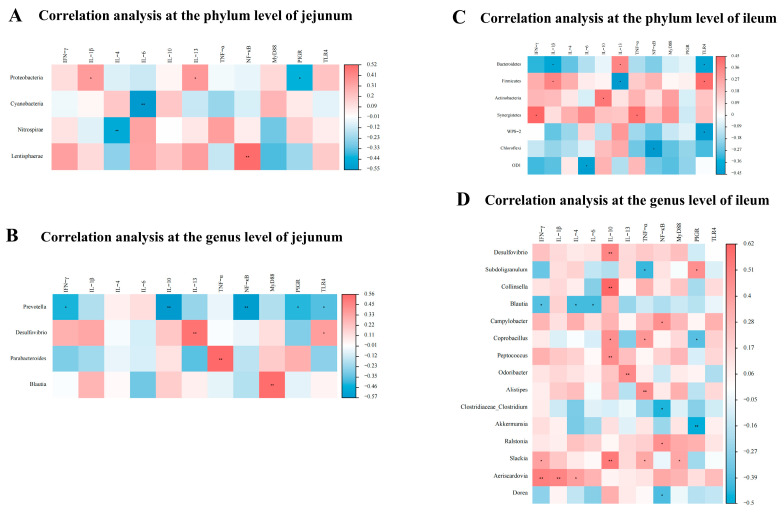
Spearman’s correlation analysis between abundant cecal microbiota and intestinal-barrier-function-related gene expressions in the jejunum (**A**,**B**) and ileum (**C**,**D**) of laying hens at the phylum (**A**,**C**) and genus (**B**,**D**) levels (*n* = 8). Red represents a significant positive correlation, and blue represents a significant negative correlation. * *p* < 0.05, ** *p* < 0.01, R > 0.4. *IFN-γ*, interferon gamma; *IL-1β*, interleukin 1 beta; *IL-4*, interleukin 4; *IL-6*, interleukin 6; *IL-10*, interleukin 10; *IL-13*, interleukin 13; *MyD88*, myeloid differentiation factor 88; *NF-κB*, nuclear factor kappa-B; *PIGR*, polymeric immunoglobulin receptor; *TNF-α*, tumor necrosis factor alpha; *TLR4*, toll-like receptor 4.

**Figure 10:**
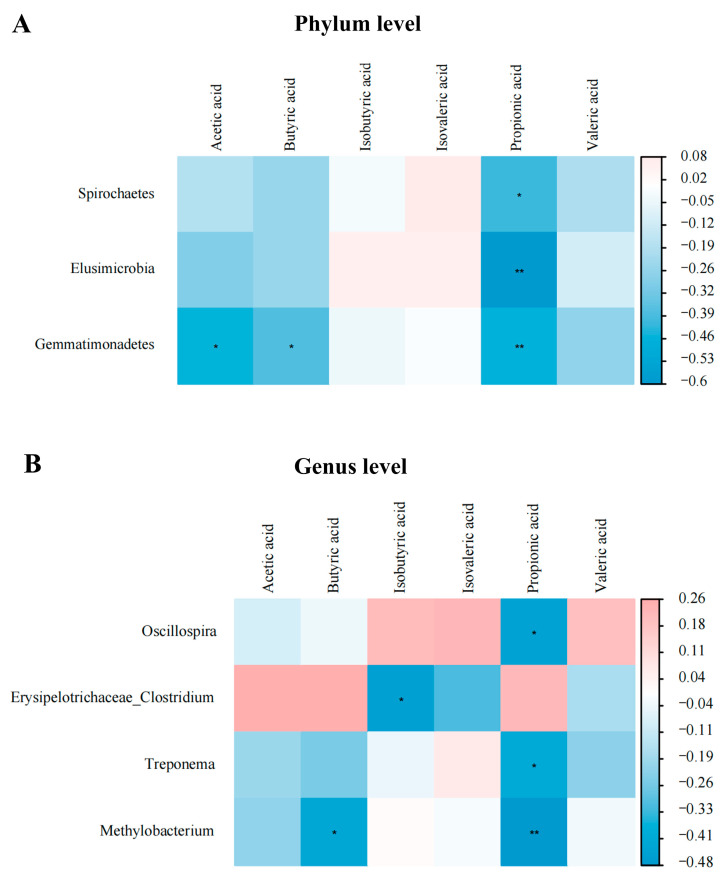
Spearman’s correlation analysis between short-chain fatty acid concentrations and abundant microbiota at the phylum (**A**) and genus (**B**) levels (*n* = 8). Red represents a significant positive correlation, and blue represents a significant negative correlation. * *p* < 0.05, ** *p* < 0.01, R > 0.4.

A Spearman’s correlation analysis was employed to determine the correlations among the main bacterial abundances, intestinal-barrier-function-related gene expressions, and SCFA concentrations (Figure 9 and Figure 10). As shown in Figure 9A,B, jejunal *NF-κB* expression was positively correlated with *Lentisphaerae* abundance, while it was negatively correlated with *Prevotella* abundance (*p* < 0.05). Additionally, jejunal *MyD88* expression was positively correlated with *Blautia* abundance (*p* < 0.05). As shown in Figure 9C,D, ileal *IL-4* expression was positively correlated with *Aeriscardovia* abundance, while negatively correlated with *Blautia* abundance (*p* < 0.05). In addition, ileal *IL-10* expression was positively correlated with *Actinobacteria*, *Desulfovibrio*, *Collinsella*, *Coprobacillus*, *Peptococcus*, and *Slackia* abundances (*p* < 0.05). As shown in Figure 10A,B, acetic acid concentration was negatively correlated (*p* < 0.05) with *Gemmatimonadetes* abundance, as well as butyric acid concentration with *Gemmatimonadetes and Methylobacterium* abundances. Moreover, isobutyric acid concentration was negatively correlated (*p* < 0.05) with *Erysipelotrichaceae_Clostridium* abundance, as well as propionic acid concentration with *Spirochaetes*, *Elusimicrobia*, *Gemmatimonadetes*, Oscillospira, *Treponema*, and *Methylobacterium* abundances.

## 4. Discussion

Intestinal barriers, including mechanical, chemical, immune, and microbial barriers, cooperatively fight against the invasion of bacteria and toxins through different molecular regulatory mechanisms and signaling pathways in animals. Thus, their integrity is of great significance for maintaining the intestinal homeostasis and body health of animals. Therefore, the present study aimed to investigate the effects of FBP on the intestinal barrier function and cecal microbiome of laying hens during the late laying period. Our findings indicated that dietary FBP supplementation improved the morphology and barrier functions of the jejunum and ileum and increased the abundance of *Prevotella* in the cecum.

Intact intestinal villi can effectively prevent the invasion of pathogenic microbes and toxins. Thus, the morphology of an intestine can directly reflect the function of its intestinal barrier. VH, CD, VCR, and VSA are the main indicators used to measure intestinal morphology. VH reflects the digestion and absorption capacity of nutrients in the small intestine [15], while VCR and VSA comprehensively reflect the development of the small intestine and the state of its digestive and absorbable function [16]. In the present study, VH in the jejunum and ileum of the 0.25–0.5% FBP groups and VSA in the jejunum of the 0.25% FBP group were higher, indicating that dietary FBP improved intestinal morphology, thus enhancing the nutrient absorption function of laying hens. Moreover, the morphological structure of the jejunum and ileum of the FBP groups was more intact and regularly shaped than that of the control group. A previous study reported that dietary 4% and 8% FBP supplementation relieved the damage to small intestinal morphology resulting from a high-fat diet by increasing the VH and VCR [17], which is consistent with the present study. It is postulated that dietary FBP may improve intestinal morphology by regulating the balance of intestinal microbiota and microbial metabolites and promoting the proliferation and differentiation of intestinal epithelial cells. However, the specific mechanism still needs to be elucidated.

The intestinal mechanical barrier, including mucosal epithelial structures and tight junctions between epithelial cells, is the first line of defense against pathogens and toxic substances [18]. Tight junctions mainly comprise claudins, occludins, and ZO-1 proteins [19]. Both occludins and claudins play a fundamental role in maintaining the physiological structures of tight junctions and regulating intestinal permeability, while ZO-1 has been shown to be imperative for tight junctions and anchoring [20]. In the present study, dietary 1.0% FBP supplementation downregulated the expressions of physical-barrier-function-related genes, including *cadherin-1*, *claudin-1*, *occludin*, and *ZO-1* in the ileum, indicating that a higher dose of FBP could damage the integrity of the tight junctions and physical barriers in the ileum of laying hens. Similarly, a previous study found that blueberry supplementation to *Escherichia coli* alleviated membrane permeability damage by upregulating the expressions of tight-junction-related genes, including *occludins* and *claudins* [21]. Anthocyanins have antimicrobial and antiadhesive properties [22]. Mo et al. [23] demonstrated that mulberry anthocyanins restored tissue structure and intestinal integrity changes by upregulating tight junction protein expressions. However, the anthocyanin content of blueberry pomace decreased after fermentation, leading to a decrease in its bioactivity [24]. In addition, the cellulose presented in FBP may increase the burden on the intestines, which might be one of the possible reasons for the damaged tight junction of the ileum in laying hens in the present study. However, further investigations are needed to elucidate the exact mechanism.

The chemical barrier is composed of mucus and digestive fluid secreted by intestinal epithelial cells and bacteriostatic substances secreted by normal bacteria. Mucin-2 is released from goblet cells and forms the backbone of the mucous layer by forming a polymer network [25]. Therefore, mucin-2 plays a key role in the chemical barrier. In the present study, *mucin-2* expression was upregulated in the jejunum of the 0.5% FBP group and the ileum of the 0.25–0.5% FBP groups. These findings indicate that dietary FBP supplementation increased the stability of the intestinal mucous layer, thereby improving the intestinal chemical barrier function of laying hens. Consistent with these findings, a previous study also reported that dietary blueberry powder supplementation upregulated *mucin-2* expression in the ileum of high-fat rats [26]. Additionally, supplementing polyphenols and proanthocyanidins can enhance intestinal digestive and defensive functions by inducing the expression of *mucin-2* in animal models [25]. Consequently, it can be speculated that the enhanced chemical barrier in the present study is due to the abundant polyphenols and proanthocyanidins present in FBP.

The intestinal immune barrier is composed of gut-associated lymphoid tissue, which secretes immunoglobulins and cytokines. Additionally, the intestinal immune barrier plays a crucial role in combating intestinal infections and maintaining intestinal environmental homeostasis [27]. Cytokines are generally divided into pro-inflammatory (including TNF-α, IFN-γ, and IL-1β) and anti-inflammatory (including IL-4, IL-10, and IL-13) cytokines. When the secretion of pro-inflammatory cytokines and the permeability of the intestinal epithelium increases, the body’s inflammatory immune response is activated [28]. The inflammatory response is mainly mediated by the TLR4-MyD88-NF-κB signaling pathway. The interaction between TLR4 and lipopolysaccharide activates a signaling cascade mediated by MyD88, which in turn triggers the activation of the NF-κB pathway [29,30]. Then, the activation of NF-κB increases the synthesis of pro-inflammatory factors, ultimately leading to the occurrence of inflammation [31]. In the present study, dietary 1.0% FBP supplementation upregulated the *IL-4* and *IL-13* expressions in the ileum of laying hens, indicating that FBP enhanced the immune function of laying hens during the late laying period. Additionally, dietary FBP supplementation downregulated the *MyD88* and *NF-κB* expressions in the jejunum of laying hens, indicating that FBP suppressed the occurrence of inflammation. Cheng et al. [32] also suggested that dietary FBP supplementation to high-fat mice downregulated the expressions of pro-inflammatory cytokines (such as *TNF-α*), ultimately alleviating inflammation in mice. These findings indicate that the intestinal immune barrier function of laying hens improved through different regulatory pathways. Possible explanations for the discrepancy may be due to the variances in animal models (high-fat mice and laying hens) and bacteria used for fermentation (*Lactobacillus casei* and microbial agents made by mixing *Bacillus subtilis*, *B. licheniformis*, *L. plantarum*, and marine red yeast).

The Spearman’s correlation analysis between intestinal-barrier-function-related gene expressions and cecal microbiota abundances revealed that jejunal *NF-κB* expression was negatively correlated with *Prevotella* abundance. The potential correlation of microbiota abundances may be explained by the fact that dietary FBP downregulated the jejunal *NF-κB* expression by increasing the abundance of *Prevotella.* Zhu et al. [33] reported that the targeted reduction of *Enterobacteriaceae* alleviated inflammation in a mouse model of colitis by inhibiting molecular-dependent critical enzymes. These findings suggest that the enhancement of intestinal immune function may be related to the species and abundance of microbiota.

The intestinal microbiota participates in physiological processes such as metabolism and immune defense and plays an important role in the intestinal health of animals [34]. The diversity of intestinal microbiota represents the complexity of microbes in terms of species, number, and functional activity, and a higher diversity generally means a healthier gut environment. In the present study, no significant differences were found in the alpha-diversity of microbiota between the control and FBP groups. A previous study reported that dietary FBP supplementation increased microbiota diversity and richness by increasing the Shannon index and decreasing the Simpson index of the fecal microbiota of high-fat mice [35]. The possible reason for the difference between the present study and previous studies is that different experimental animals have different absorption and utilization capacities of FBP. The beta-diversity analysis indicated that the cecal microbial communities between the control and FBP groups were distinctly separated, indicating that dietary FBP significantly changed the cecal microbiota composition of laying hens during the late laying period.

*Firmicutes* and *Bacteroidetes* were the two dominant phyla in the cecum of laying hens in the present study, which is consistent with a previous report [36]. *Prevotella*, one of the fundamental genera of intestinal bacteria, inhabits the gastrointestinal tract, oral cavity, and skin [37]. *Prevotella* can decompose proteins and carbohydrates, and its main metabolites (including acetic acid, isobutyric acid, and isovaleric acid) enhance intestinal health. However, *Prevotella* is a double-edged sword. Increases or decreases in the abundance of *Prevotella* are related to several diseases, such as rheumatoid arthritis and multiple sclerosis [38,39]. In the present study, dietary 0.5–1.0% FBP supplementation increased the abundance of *Prevotella* in the cecum of laying hens, indicating that dietary FBP maintained the homeostasis of the intestinal microbiota by increasing the abundance of beneficial bacteria, thereby improving the intestinal health of laying hens. However, there is currently no relevant research on the effect of FBP on the gut microbiota of laying hens, which warrants further research to explore the specific mechanisms.

*Olsenella* has been shown to have better antitumor immune effects and anti-CTLA-4 efficacy than *Colidextribacter* species in a murine tumor model [40]. In addition, it is speculated that the acidogenic bacteria *Petrimonas* may help to optimize the composition of SCFAs and drive the formation of acetic acid [41]. In the present study, dietary 0.5–1.0% FBP supplementation enriched beneficial bacteria such as *Olsenella*, *Prevotella*, and *Petrimonas* in the cecum of laying hens during the late laying period, indicating that FBP improved gut microbiota composition in the laying hens.

Glutamine and aspartate can be metabolized to alanine, which is involved in the synthesis and metabolism of proteins, glycogen, and fatty acids in the body [42]. Methane metabolism plays an important role in the global carbon cycle and climate change [43]. In the present study, a predicted functional profile analysis showed that FBP supplementation enhanced several metabolic functions, such as alanine/aspartate/glutamate metabolism and methane metabolism in the cecum of laying hens, indicating that FBP promoted amino acid and methane metabolism. However, further in-depth studies are necessary to elucidate how FBP affects amino acid and methane metabolism.

Acetic acid and propionic acid are mainly produced by *Bacteroides*, while butyric acid is mainly produced by *Firmicutes* [44]. SCFAs are the main energy source for intestinal epithelial cells and can also enhance the mucosal barrier and regulate inflammation [45]. In the present study, there were no significant differences in the SCFA concentrations between the control and FBP groups. A previous study demonstrated that blueberry supplementation to rats improved epithelial barrier function by increasing the concentrations of SCFAs [46]. Moreover, a recent study also indicated that grape pomace supplementation to broiler chickens had no effect on the concentrations of SCFAs in the cecum [47]. SCFAs are quickly absorbed and utilized by the gut or other bacteria [48]. Therefore, the concentrations of SCFAs are related to the metabolic rate of the animal, which may be the reason for the differences. Although dietary FBP had no significant effects on SCFA concentrations, a further Spearman’s correlation analysis showed that acetic acid was negatively correlated with *Gemmatimonadetes* abundance, as well as butyric acid concentration with *Gemmatimonadetes* and *Methylobacterium* abundances. Moreover, isobutyric acid had a significant negative correlation with the abundance of *Erysipelotrichaceae_Clostridium*, as well as propionic acid with the abundances of *Spirochaetes*, *Elusimicrobia*, *Gemmatimonadetes*, *Oscillospira*, *Treponema*, *and Methylobacterium.* Polysaccharides increased *Peptoclostrium* abundance, and polyphenols increased *Clostridium* and *Lachnochlostrium* abundances, both of which together increased the total concentrations of fecal SCFAs in high-fat-fed mice [49]. In the present study, dietary FBP supplementation did not significantly increase the abundances of *Peptoclostrium*, *Clostridium*, and *Lachnochlostrium*, resulting in no significant increase in the cecal SCFA concentrations. More research is needed to explore the reasons why FBP has no significant difference in these three microbiota abundances.

## 5. Conclusions

In summary, dietary FBP supplementation improved the digestive and absorption capacity of the intestine for nutrients by increasing VSA in the jejunum and VH in the jejunum and ileum of laying hens during the late laying period. Additionally, FBP supplementation improved intestinal barrier function by upregulating the expressions of *mucin-2*, *IL-4*, and *IL-13* and increasing *Prevotella* abundance. Our results indicated that 0.25 to 0.5% FBP had a better effect than 1.0% FBP. These findings provide a reference for the utilization of FBP to improve the intestinal health of laying hens during the late laying period.

## Figures and Tables

**Table 1:** The composition and nutrient levels of the basal diet (%, as feed basis).

Item	Content
Composition	
Corn	64.20
Soybean meal	21.60
Limestone	8.00
Soybean oil	1.20
Premix ^1^	5.00
Total	100.00
Nutrient level ^2^	
Crude protein	15.56
Crude fat	5.10
Metabolic energy, MJ/kg	11.38
Calcium	3.52
Phosphorus	0.42
Lysine	0.95
Methionine	0.33
Methionine + Cysteine	0.58
Tyrosine	0.43

^1^ Premix provided per kg of diet: vitamin A, 10,000 IU; vitamin D_3_, 3000 IU; vitamin E, 20 IU; vitamin K_3_, 1.75 mg; vitamin B_1_, 2 mg; vitamin B_2_, 6 mg; vitamin B_6_, 3 mg; pantothenic acid, 8.5 mg; vitamin B_12_, 0.02 mg; nicotinamide, 40 mg; folic acid, 1 mg; biotin, 0.24 mg; choline chloride, 450 mg; methionine, 1.3 g; lysine, 0.95 g; calcium, 5 g; phosphorus, 0.75 g; copper, 8 mg; iron, 75 mg; manganese, 100 mg; zinc, 65 mg; iodine, 0.8 mg; selenium, 0.3 mg; sodium chloride, 3 g. ^2^ The nutrient level is the measured value.

**Table 2:** The primer sequence of genes for RT-qPCR.

Gene Name	Accession Number	Primer Sequence (5′-3′)	Product Size (bp)
*Cadherin 1*	NM_001039258.3	F: TCGTCAGCTACTCCATCGTC R: TCGTACATGGTGGGGTTGAA	247
*Claudin 1*	NM_001013611.2	F: AGGTGTACGACTCGCTGCTT R: AGCCACTCTGTTGCCATACC	242
*Claudin 2*	NM_001277622.1	F: CCTGCTCACCCTCATTGGAG R: GCTGAACTCACTCTTGGGCT	144
*IFN-γ*	NM_205427.1	F: CTCACGCTCCTTCTGAAAGC R: GATGGTGTCGTTGAAGGAGC	171
*IL-1β*	NM_204524.2	F: TCCTCCAGCCAGAAAGTGAG R: GTCCAGGCGGTAGAAGATGA	228
*IL-4*	NM_001007079.2	F: CCCAGAAAAGCACAGACAGG R: TCTTGACGCAGGAAACCTCT	186
*IL-6*	NM_204628.2	F: TGTGCAAGAAGTTCACCGTG R: ACTCGACGTTCTGCTTTTCG	213
*IL-10*	NM_001004414.4	F: TGTACCATTTGTGGCAGTGC R: TCGTCTGGTGTTTGCAGTTG	174
*IL-13*	NM_001007085.3	F: CCGCATCCCCATACTCATCT R: GTTGATGGCTGCTTTGGGAA	207
*Mucin 2*	NM_001318434.1	F: TGTGTTTGAGAAGTGCCGTG R: AGAGCAGCAAACACCATTGG	184
*MYD88*	NM_001030962.5	F: CCACGACTACCTGGAGATCC R: ACTTCTTGCAGTCCTCCTCC	188
*NF-κB*	NM_001396038.1	F: CAACCCCTTCAATGTGCCAA R: ACCTTGTCACAGAGCAGGAA	240
*Occludin*	NM_205128.1	F: CCGTAACCCCGAGTTGGAT R: ATTGAGGCGGTCGTTGATG	214
*PIGR*	NM_001044644.2	F: CTGTGACTTCGGGGAGGATT R: GGGTGATCATGACGCTGAAC	170
*TLR4*	NM_001030693.2	F: ATGTCCTCTTGCCATCCCAA R: TCTCCCCTTTCTGCAGAGTG	158
*TNF-α*	NM_204267.2	F: GTATGTGCAGCAACCCGTAG R: TGGGCATTGCAATTTGGACA	228
*ZO-1*	XM_015278975.2	F: TTTACATGATGACCGCCTGT R: GAATCCTCCCTAACGGGTTC	213
*β-actin*	NM_205518.1	F: ATGAAGCCCAGAGCAAAAGA R: GGGGTGTTGAAGGTCTCAAA	223

*IFN-γ*, interferon gamma; *IL-1β*, interleukin 1 beta; *IL-4*, interleukin 4; *IL-6*, interleukin 6; *IL-10*, interleukin 10; *IL-13*, interleukin 13; *MyD88*, myeloid differentiation factor 88; *NF-κB*, nuclear factor kappa-B; *PIGR*, polymeric immunoglobulin receptor; *TLR4*, toll-like receptor 4; *TNF-α*, tumor necrosis factor alpha; *ZO-1*, zonula occludens-1.

**Table 3:** Effects of fermented blueberry pomace (FBP) on the jejunal and ileal morphology of laying hens during the late laying period ^1^.

Items ^2^	Dietary Groups				SEM	*p*-Values
	Control	0.25% FBP	0.5% FBP	1.0% FBP		
Jejunum						
CD, μm	193.51	235.31	253.77	252.33	11.00	0.178
VCR	5.62	6.56	5.54	5.31	0.28	0.432
VH, μm	1017.24 ^b^	1435.85 ^a^	1397.85 ^a^	1230.73 ^ab^	52.53	0.004
VSA, μm^2^	344.11 ^b^	664.52 ^a^	569.51 ^ab^	416.13 ^ab^	43.07	0.015
VW, μm	213.11	289.74	258.84	212.58	14.13	0.141
Ileum						
CD, μm	153.17	192.69	212.19	174.85	9.23	0.122
VCR	7.13	7.59	6.28	5.51	0.36	0.180
VH, μm	1020.28	1349.08	1278.06	935.87	67.56	0.070
VSA, μm^2^	362.61	459.86	566.30	256.14	58.68	0.297
VW, μm	216.90	215.00	256.07	169.34	15.83	0.307

^1^ Data are expressed as the mean and SEM (*n* = 4). Means within a row with different superscript letters are significantly different (*p* < 0.05). ^2^ CD, crypt depth; VH, villus height; VCR, VH to CD ratio; VSA, villus surface area; VW, villus width.

**Table 4:** Effects of fermented blueberry pomace (FBP) on the cecal short-chain fatty acids of laying hens during the late laying period ^1^.

Items, μmol/g	Dietary Groups				SEM	*p*-Values
	Control	0.25% FBP	0.5% FBP	1.0% FBP		
Acetic acid	74.90	71.39	81.90	83.85	2.50	0.256
Butyric acid	5.24	5.90	7.63	7.54	0.50	0.240
Isobutyric acid	3.62	2.76	3.27	4.02	0.22	0.200
Isovaleric acid	1.50	1.30	1.34	1.66	0.09	0.467
Propionic acid	20.83	22.88	25.71	25.00	1.04	0.350
Valeric acid	3.08	2.50	2.88	3.69	0.19	0.166

^1^ Data are expressed as the mean and SEM (*n* = 8).

## Data Availability

The data presented in this study are openly available in the Science Data Bank at https://www.scidb.cn/s/jyeYRz, accessed on 5 September 2024.
