# Correction: Qin et al. Dietary Fermented Blueberry Pomace Supplementation Improves Small Intestinal Barrier Function and Modulates Cecal Microbiota in Aged Laying Hens. *Animals* 2024, *14*, 2786

**DOI:** 10.3390/ani15121793

**Published:** 2025-06-18

**Authors:** Binghua Qin, Zhihua Li, Qian Zhu, Ting Chen, Wei Lan, Yadong Cui, Md. Abul Kalam Azad, Xiangfeng Kong

**Affiliations:** 1 Key Laboratory of Agro-Ecological Processes in Subtropical Region, Hunan Provincial Key Laboratory of Animal Nutrition Physiology and Metabolic Processes, Institute of Subtropical Agriculture, Chinese Academy of Sciences, Changsha 410125, China; qinbinghua23@mails.ucas.ac.cn (B.Q.); zhli92@126.com (Z.L.); zhuqian@isa.ac.cn (Q.Z.); chenting23@mails.ucas.ac.cn (T.C.); 2 School of Biology and Food Engineering, Fuyang Normal University, Fuyang 236037, China; lanwei@fynu.edu.cn (W.L.); fysyswx@163.com (Y.C.); 3 College of Advanced Agricultural Sciences, University of Chinese Academy of Sciences, Beijing 100049, China

Error in Table

In the original publication [1], there was an error in Table 1 as published. The metabolic energy 15.14 MJ/kg, should be 11.38 MJ/kg. The corrected Table 1 appears below. The authors state that the scientific conclusions are unaffected. This correction was approved by the Academic Editor. The original publication has also been updated.

## Figures and Tables

**Table 1:** The composition and nutrient levels of the basal diet (%, as feed basis).

Item	Content
Composition	
Corn	64.20
Soybean meal	21.60
Limestone	8.00
Soybean oil	1.20
Premix ^1^	5.00
Total	100.00
Nutrient level ^2^	
Crude protein	15.56
Crude fat	5.10
Metabolic energy, MJ/kg	11.38
Calcium	3.52
Phosphorus	0.42
Lysine	0.95
Methionine	0.33
Methionine + Cysteine	0.58
Tyrosine	0.43

^1^ Premix provided per kg of diet: vitamin A, 10,000 IU; vitamin D_3_, 3000 IU; vitamin E, 20 IU; vitamin K_3_, 1.75 mg; vitamin B_1_, 2 mg; vitamin B_2_, 6 mg; vitamin B_6_, 3 mg; pantothenic acid, 8.5 mg; vitamin B_12_, 0.02 mg; nicotinamide, 40 mg; folic acid, 1 mg; biotin, 0.24 mg; choline chloride, 450 mg; methionine, 1.3 g; lysine, 0.95 g; calcium, 5 g; phosphorus, 0.75 g; copper, 8 mg; iron, 75 mg; manganese, 100 mg; zinc, 65 mg; iodine, 0.8 mg; selenium, 0.3 mg; sodium chloride, 3 g. ^2^ The nutrient level is the measured value.
